# Unveiling the need of interactions for social N400s and supporting the N400 inhibition hypothesis

**DOI:** 10.1038/s41598-023-39345-6

**Published:** 2023-08-03

**Authors:** Sujata Sinha, Sarah Del Goleto, Milena Kostova, J. Bruno Debruille

**Affiliations:** 1 Department of Neurosciences, Faculty of Medicine, McGill University, Montréal, Canada; 2 Research Center of the Douglas Mental Health University Institute, Montréal, Canada; 3 UR Paragraphe, Université Paris 8 Vincennes-Saint-Denis, Saint-Denis, France; 4 Department of Psychiatry, Faculty of Medicine, McGill University, Montréal, Canada

**Keywords:** Neuroscience, Cognitive neuroscience, Social neuroscience

## Abstract

When participants (Pps) are presented with stimuli in the presence of another person, they may consider that person’s perspective. Indeed, five recent ERP studies show that the amplitudes of their N400s are increased. The two most recent ones reveal that these social-N400 increases occur even when instructions do not require a focus on the other's perspective. These increases also happen when Pps know that this other person has the same stimulus information as they have. However, in all these works, Pps could see the other person. Here, we tested whether the interaction occurring with this sight is important or whether these social N400 increases also occur when the other person is seated a bit behind Pps, who are aware of it. All had to decide whether the word ending short stories was coherent, incoherent, or equivocal. No social N400 increase was observed: N400s elicited by those words in Pps who were with a confederate (n = 50) were similar to those of Pps who were alone (n = 51). On the other hand, equivocal endings did not elicit larger N400s than coherent ones but triggered larger late posterior positivities (LPPs), like in previous studies. The discussion focuses on the circumstances in which perspective-taking occurs and on the functional significance of the N400 and the LPP.

## Introduction

Our social interactions require the ability to consider the perspective of the person we are dealing with, to be aware of the information that is shared and to base our expression on this common ground. Hundreds of studies have already been done on these abilities (see reviews^1–4^). However, many of them focus on information that are easily verbalizable. Yet, during the initial contact, a great deal of information is exchanged in a way that cannot be easily verbalized, such as the impression left by the attire, posture, facial expression, gaze and gestures together with the apparent age and sex of the person we are meeting. Recording the brain activity that occurs during such a visual contact could go some way to filling this gap. This is shown by recent studies of one component of this activity, the N400 event-related brain potential (ERP). Indeed, this ERP has already been examined when it was evoked by a stimulus perceived by 2 people in visual interaction. However, before turning to the results of these studies, we will briefly summarize some of the main findings about the N400. The aim is to approach its functional significance from the results obtained in isolated participants first.

The N400 is a negative-going event-related brain potential (ERP) characterized by its maximal voltage around 400 ms after the presentation of meaningful stimuli, such as words, faces, objects and scenes^5–13^. It is usually observed during tasks that do not distract participants too much from semantics. So, it is not seen in tasks that focus on physical features, such as deciding whether the words used as stimuli are written with upper- or lower-case letters^14,15^. The N400 has thus been extensively studied to understand the semantic processing of words, both of isolated ones and of those occurring within different types of sentence contexts. As could have been expected, words with little meaning, such as closed-class words, like “the,” were found to elicit much smaller N400 potentials than meaningful words, like “apple”^16,17^. Most importantly, when a word is unexpected in the context in which it appears, such as the word “honey” in the sentence: “He takes his coffee with cream and honey,” hundreds of studies have demonstrated that it will elicit a larger N400 response than when the meaning of this word is primed (e.g.^5,6,18^).

The N400 has thus been proposed to index the access to, the retrieval of, or the early activation of the semantic representations of the stimulus that were not primed by previous stimuli (e.g.^19^; for a review see^6^). However, some studies (e.g.^20,21^) reported that cortical activations for real words differed from those elicited by matched pseudo-words (e.g., toble) earlier than the onset the N400 (i.e., earlier that 250 ms). The access to lexical representations, and thus to semantics, is therefore likely to occur before the N400. Partly because of the lateness of its onset and of the maximum of its voltage, the N400 has also been suggested to index the late integration of these activated representations within the representation of the context (e.g.,^22^; for debates, see for instance^23–25^). On the other hand, detailed sets of relations between the N400 amplitude and predictive errors have been developed more recently (e.g.,^26–28^). Models developed from these relations accurately predict N400 amplitudes in a variety of settings (for a review, see ^28^).

Five recent social studies have shown that the presence of another person, such as that of a confederate, can have an impact on the N400. The first three studies^29–31^ have reported increases of the amplitude of this ERP when two particular conditions are met. First, when participants have all the information necessary to understand the stimulus but know that some information is missing to the other person. Second, when these participants are asked whether the stimulus makes sense not only to them but also to this person, and thus, are asked to take also the other’s perspective. This N400-increase, now known as the “social-N400 effect”^29^, was interpreted as indexing the difficulty at integrating the meaning of the stimuli when a piece of the priming information is missing. Accordingly, participants would mirror the integration difficulty of the confederate who is missing that piece. This interpretation of the social-N400 effect thus combines the N400-integration difficulty hypothesis with mentalization, or theory of mind, processes (e.g.,^32,33^). It focuses on the mentalization processes that are at work to focus on the knowledge that is shared in order to maintain a common ground between two individuals^34^. The first three social N400 studies thus suggest that manipulating the common ground by hiding information from the confederate can be responsible for social-N400 effects.

This manipulation of the common ground requires the participant to set aside the privileged information (s)he had, as detailed in^31^. Interestingly, the results obtained in the three social N400 studies mentioned could then also be interpreted using a N400 hypothesis that has not been introduced yet. This hypothesis stipulates that this ERP indexes processes that occur between the activations and the late integration and that inhibit inappropriately activated information, such as inaccurate predictions (for a review see^35^; for data see^36–40^, for discussion see supplementary material). These inhibitory processes might then be the ones by which the privileged information is set aside to be able to focus on the common ground.

In the first task of their second experiment, Jouravlev et al.^31^ found that the presence of a confederate also leads to an increase of the N400 amplitude when participants are simply asked if the stimulus made sense, thus *not* mentioning the confederate in the instructions. Nevertheless, this increase was not found in a task that was a bit more difficult. There, participants had to decide, for example, whether Mary is a vegetarian chef after seeing: “Mary is making an unusual dessert from bacon. Mary sprinkled the bacon with sugar and nutmeg”^31^. Taken together, these results suggest that the social-N400 effect may be observed only when the task is not too difficult. Namely, when it allows the participant to take the perspective of the confederate in addition to his/her own. This raises the possibility that this perspective-taking occurs spontaneously and is an automatic phenomenon which may be prevented in some circumstances. Indeed, an important part of perspective-taking is considered as belonging to theory-of-mind (ToM) processes that can occur without our conscious control (for a review, see^41^). It can thus be seen as an implicit form of mentalization process (e.g.,^42^). Nevertheless, although perspective taking can happen spontaneously, somehow like other automatic processes, it might not occur systematically. Like the knee reflex, for instance, it might be suppressed, or boosted. This automaticity and adjustability are also probably true for the particular part of the perspective-taking that modulates N400 amplitude. In effect, N400 processes appear even during attentional blinks^43^, which supports their automaticity, and they are also modulated by task instructions, as they are reduced to a minimum by tasks that focus on physical features (e.g., deciding whether word letters are upper- or lower-cases) and boosted to a maximum in semantic tasks^14^, which supports their adjustability.

Using again a task that was not too difficult (i.e., a sentence correctness task) and that did not explicitly require any focus on the perspective of the other person, the authors of the fourth recent study^44^ found that there was no need of presenting more information to the participant than to the other person. They reported that a social N400 increase was induced by the mere presence of such a person when both are exposed to the same stimuli at the same time, bringing further support to the automaticity of perspective taking. The results obtained by Forgács et al.^45^ (the fifth recent study) confirmed this finding. In their first two experiments, the mere presence of another person was again found to induce an N400 increase. There, both the participant and the other person were also perceiving all the stimulus material and had the same information. Moreover, there was a complete absence of any task instruction. Forgacs et al.^45^ interpret the social N400 increase as an index of the retrieval of the meanings of stimuli “on behalf of, for and by the social partner” to incorporate the partner’s mental state and belief. Taken together, these results thus suggest that the perspective-taking spontaneously occurs in situations that are comparable to those of everyday life where two (or more) persons witness together the same event. They also reveal that manipulating the common ground by hiding some information is not necessary for observing a social N400.

Interestingly, in the five studies mentioned^29–31,44,45^, the social N400 effects found were observed in participants who could see the confederate during the entire experiment. In effect, in four of these studies^29–31,44^, this confederate was sitting next to them. (S)he was therefore constantly in the periphery of their visual field and were in full sight when participants were turning their head and/or their eyes. In the last one^45^, the confederate was facing the participant. Being seen and seeing another person is, as mentioned at the very beginning of this introduction, a social interaction. It thus probably triggers the building of a common ground even when participants are not talking to each other.

This building should imply perspective taking, which can occur even if no information is hidden from the confederate by the experimenter. Indeed, meaningful stimuli also activate representations that are specific to the participant, such as representations that correspond to personal contexts, emotions and/or affordances. For instance, the stimulus word "cup" may activate contexts in which the participant used this object, heard the word or read it before the experiment. This constitutes privileged information that may also have to be set aside.

The larger N400s obtained in the studies by Hinchcliffe et al.^44^ and Forgacs et al.^45^ in the presence of another person than alone, could thus be due to the same causes as those at work in the studies by Rueschmeyer et al.^29^ and Jouravlev et al.^31^ Namely, they could be due to the building of a common ground when interacting with someone. If this were the case, then, making interaction impossible by placing the confederate out of the sight of the participant might prevent this building and make N400 amplitudes similar to those obtained without a confederate.

On the contrary, larger N400s might be observed even when the confederate is out of sight, for instance, when (s)he is behind participants. This would then mean that some common ground building occurs, automatically, just when participants know they are in the presence of someone else, witnessing the same events, even if no interaction occurs between them. Participants may also process and store the stimulus from the position of the confederate too, binding it to the representations of this person. In effect, visual perspective taking, for instance, can produce ERP effects in alone conditions (e.g.,^46^).

The goal of the current study was thus to examine this alternative and therefore to further specify the circumstances of occurrence of the common ground building indexed by social N400s. The absence of ERP difference between participants with- and those without-a confederate would strengthen the first possibility whereas their presence would support the second one.

To achieve this goal, we looked not only at raw ERPs but also at ERP effects, particularly at the classical N400 effect that is observed between coherent/predictable stimuli and incoherent/unpredictable ones^5,6,18^. To do so, a design similar to that of Del Goleto et al.’s study^47^ was chosen because it includes these two types of stimuli and because it also offers the possibility to further test the N400 inhibition hypothesis. Indeed, this design also has equivocal stimuli that have to be responded to as such. This requires participants to take into account their relationship with the coherent/predicted stimulus, which should prevent its inhibition. Equivocal stimuli could thus, despite having lower predictability and inducing greater integration difficulty than coherent words, elicit N400 amplitudes similar to those evoked by predicted/coherent stimuli. Indeed, the inhibition hypothesis predicts an absence of larger N400s when there is no additional inhibition. This appears to be what was already observed in^47–49^ where the equivocality of some words, relative to the expected/non equivocal ones, was task relevant. This could, in turn, help to understand the functional significance of social N400s. Increased N400s might index the processes by which privileged information is set aside.

## Methods

### Participants

Participants were recruited by ads placed for research course credit for the students of the psychology department of the Université de Vincennes, Paris 8. Potential participants had to have either normal or corrected-to-normal vision. Those who had history of mental disorders and those who were consuming psychotropic drugs more than once a week were excluded using the Mini International Neuropsychiatric Interview^50^ (the MINI). They had to fill-up the Edinburgh Handedness Inventory^51^ and the French version of the National Adult Reading Test^52^ (fNART) to evaluate their verbal intelligence.

A between-subject design was chosen to prevent a persistence of a strategy from one social context to the other. Two groups of healthy participants were thus recruited. Fifty-three of them were enrolled to do the task alone. They were then called the alones. Two of them were rejected later as they had too many trials with poor EEG (see “Data processing and measures” in the “Methods” section). Fifty-one healthy participants were recruited to do the experimental task with a confederate. They will be designated here as Participants with a Confederate (PwCs). One of them was later rejected for poor EEG. The role of the confederate was played by an experimenter who was always of the same sex as the participant. The sociodemographic data of the two groups are presented in Table 1 below.

**Table 1:** Sociodemographic data of the two groups of participants.

Sociodemographic parameters	Alones	Participants with a confederate (PwCs)
Mean (SD) age (in years)	24.7 (7.4)	23.6 (6.8)
Sex (females/males)	40/11	41/9
Mean (SD) number of years of education	14.5 (1.4)	14.3 (1.4)
Mean (SD) right-handedness score (Edinburgh laterality)	64.3 (24)	75.9 (18.9)
Mean (SD) verbal intelligence score (fNART total)	25.9 (5.9)	25 (5.4)
Mean (SD) state-anxiety score before the experiment (STAI Y-A)	31.7 (7.2)	31.5 (7.0)
Mean (SD) state-anxiety score after the experiment (STAI Y-A)	29.8 (6.8)	28.5 (6.6)

### Consent

All participants read and signed an informed consent form (included in Supplementary material, Fig. S1), which was in accordance with the Declaration of Helsinki. This form was accepted and the experiment was approved by the institutional review board of Paris-8 University.

### Stimuli

For each participant, stimuli were one hundred and eighty short stories, each featuring a main and a secondary character. Each story consisted of three sentences. The first two sentences set up the context and the last word of the third sentence, called the target word, set up the meaning of the story. The stories were either coherent, incoherent, or equivocal. Coherent stories were logical, appropriate and literal. The incoherent ones comprised stories that did not make sense. The equivocal condition contained stories that had an ambiguous meaning, which was either ironic, humorous or lying. Importantly, in itself, the last sentence was always coherent and non-ambiguous.

Each story came from an irony-, a humor- or a lie-corpus. Each of these three corpora comprised sixty sets of stories. Each of these sets consisted of three versions of the same story: a coherent, an incoherent and an equivocal version. The sets of stories of the irony corpus were taken from the verbal material of the study by Del Goleto et al.^47^. The sets of stories of the humor corpus, were selected from the verbal material of Kostova et al.’s study^53^. For the lie corpus, sixty story sets of were built on the model of the irony and humor corpora. Table 2 below provides an example of a story set for each of the three corpora.

**Table 2:** Examples of stimuli of coherent, incoherent, and equivocal (irony, humor and lie) conditions.

	Coherent stories	Incoherent stories	Equivocal stories
Corpus: irony	Pierre n’a rien mangé de la journée Pour le dîner sa mère a préparé des spaghettis Pierre dit: *je suis vraiment content!*	Pierre est en retard pour le dîner Il appelle sa mère pour s’excuser Pierre dit: *je suis vraiment content!*	Pierre a toujours détesté le poisson Pour le dîner sa mère a préparé du saumon Pierre dit: *je suis vraiment content!*
English translation of the above	Peter has not eaten anything all day For dinner his mother prepared spaghetti Peter says: *I'm really happy!*	Peter is late for dinner He calls his mother to apologize Peter says: *I'm really happy!*	Peter has always hated fish For dinner his mother prepared salmon Peter says: *I'm really happy!*
Corpus: humor	Lors d’un dîner entre amis, Eric dit: j’ai remarqué que quand on a des grandes mains on a souvent *aussi* *de gros doigts*	Lors d’un dîner entre amis, Eric dit: je pense que quand on a une famille nombreuse on a souvent *aussi de gros doigts*	Lors d’un dîner entre amis, Eric dit: je pense que les gorilles ont de grosses narines parce qu’ils ont *aussi de gros doigts*
English translation of the above	At a dinner with friends, Eric says: I noticed that when you have big hands you often *also have* *big fingers*	At a dinner with friends, Eric says: I think that when you have a large family you often *also have* *big fingers* too	At a dinner with friends, Eric says: I think that gorillas have big nostrils because they *also have big fingers*
Corpus: lie	Victor appelle son père à la sortie des cours Son père, qui l’attend à la maison, lui demande où il est Victor répond: *je sors du lycée*	Victor est à la cave pour chercher du pain dans le congélateur Son père lui demande ce qu’il fait en bas Victor répond: *je sors du lycée*	Victor a séché les cours pour aller au cinéma Quand il rentre chez lui, son père lui demande où il était Victor répond: *je sors du lycée*
English translation of the above	Victor calls his father after school His father, who is waiting for him at home, asks him where he is Victor answers: *I'm just out of school*	Victor is in the basement looking for bread in the freezer His father asks him what he is doing downstairs Victor answers: *I'm just out of school*	Victor skipped school to go to the movies When he gets home, his father asks him where he was Victor answers: *I'm just out of school*

The stimulus sequence for each participant was made of 20 coherent-story-versions, 20 incoherent-story-version and 20 equivocal-story-versions taken from each of the three corpora. Each participant was thus presented with (20 + 20 + 20) × 3 = 180 stories. Each story appeared in only one of its 3 versions. So, if a participant was presented with the story-version having a coherent ending, the two other story-versions from the same set (i.e., the incoherent and the equivocal versions) were not presented to him/her. Story-versions were counterbalanced across participants.

### Assessment of stimuli

The plausibility of the stories used, as well as the predictability of their target word, were assessed through questionnaires. Prior to the assessment, there were two hundred and three stories from the three corpora (humor, irony and lies). These stories were divided into three lists such that each list contained only one condition of each of the stories, that is, either the coherent, the incoherent or the equivocal one. These lists were further split into three sub-lists to limit the number of stories per questionnaire. One hundred and fifty-five participants completed a questionnaire of sixty-six or sixty-seven stories in which they were asked to rate the plausibility of each story on a scale of 0 to 10 (0: not at all understandable; 10: perfectly understandable). In order to have an equal number of sixty stories from each corpus, eleven stories from the lie corpus and twelve stories from the irony corpus (i.e., twenty-three stories) were withdrawn. They were the stories that had the lowest understandability in the equivocal condition. After this removal, there were thus one hundred and eighty stimulus stories for the experiment. A 2-way (Condition × List) ANOVA on the remaining one hundred and eighty stories revealed a main condition effect (F (2, 345) = 759.49; p < 0.001) with a graded understandability between the coherent, incoherent and equivocal conditions. Their means were, respectively, 8.45 (SD = 1.17), 2.26 (SD = 1.71) and 6.63 (SD = 1.57). There was no main effect of list (F < 1) nor condition × list interaction (F < 1).

Similarly, the predictability of the target word of each story was measured. Using the same breakdown into lists as above, one hundred and thirty-four different participants completed a questionnaire consisting of sixty-six or sixty-seven stories where the last word of each story was missing. The participants’ task was to write down the first word that came to mind after reading the story to fill up the missing word (the Cloze Procedure^54^). The average predictability of the last word of the coherent, incoherent and equivocal conditions were 0.53 (SD 0.35), 0.01 (SD 0.65) and 0.21 (SD 0.24), respectively. The 2-way ANOVA showed a main effect of condition (F (2, 271) = 196.3; p < 0.001) without main effect of list (F < 1) and without condition × list interaction (F < 1).

Our assessments of the stimuli thus confirmed that the coherent stories were judged more plausible than equivocal ones, which were judged more plausible than the incoherent ones. It also confirmed that the target words of the coherent stories were more predictable than those of the equivocal and incoherent stories.

### Procedure

**Figure 1:**
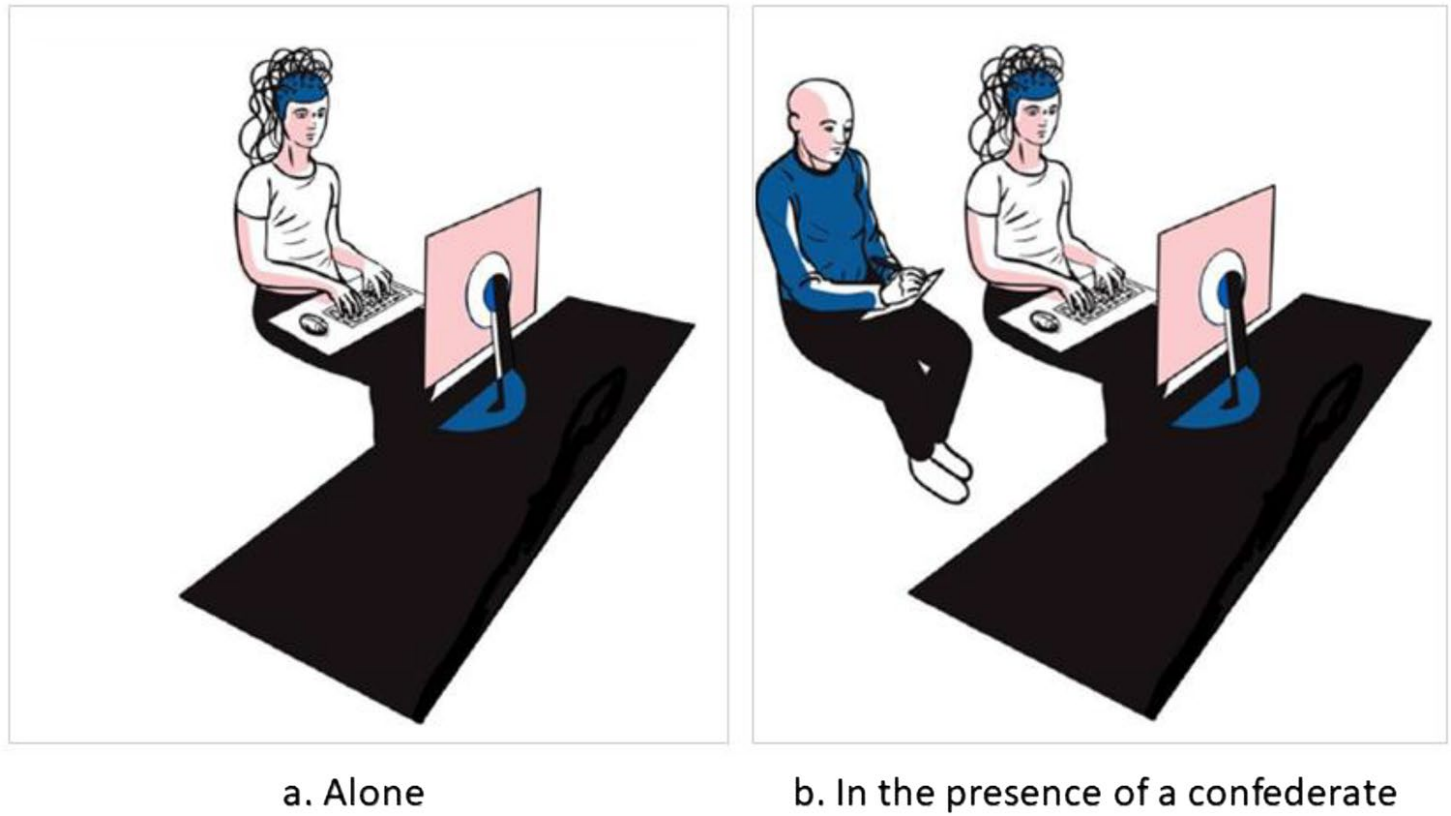
Experimental setup for the study. (**a**) Alone participants did the task alone using the keyboard. (**b**) Participants with a confederate (PwCs) did the task also using the keyboard while the confederate did it using pen and paper while seated a bit behind the participants. This prevented the participants from seeing the confederate in the periphery of their visual field.

Upon arrival at the lab, the participant was asked to fill out the informed consent form. The confederate, who arrived shortly after the participant, was taken to a separate room. (S)he was one of the trainees of the lab and did not know the participants. Given that being next to an unknown person could induce anxiety, the initial state anxiety was controlled with the state part of the State and Trait Anxiety Inventory (the STAI Y-A^55^). Participants with confederate (PwCs) were notified that the confederate would carry out the same task as them but without the EEG recording. After the EEG cap placement, the alone participants were seated alone (see Fig. 1a, below). Once the EEG cap was placed on PwCs in the experiment room, the confederate was invited and seated in a chair next to that of the participant, a little behind him/her and facing the same computer screen (see Fig. 1b, below). For both alones and PwCs, the whole experimental session consisted of a training session, the real task, which included two breaks in the middle of it, and finally, a debriefing session. Instructions were given by the experimenter to the alones and to the PwCs and their confederate. While PwCs were asked to respond to the trials by pressing a key on the keyboard, the confederate pretended to perform the task using pen and paper. Participants knew that this other person was having the same task and the same stimulus material as they were. Thus, unlike some previous social N400 studies (e.g.,^29,31^), the experimenter did not introduce privileged information. At the end of the experiment, one of the experimenters stayed with the participant for a second STAI Y-A assessment, to see if the confederate’s presence increased his/her anxiety and for the debriefing. Meanwhile, another experimenter went to another room with the confederate. No communication was allowed between the participant and the confederate during the entire duration of the experiment to prevent the participants from interacting with him/her.

**Figure 2:**
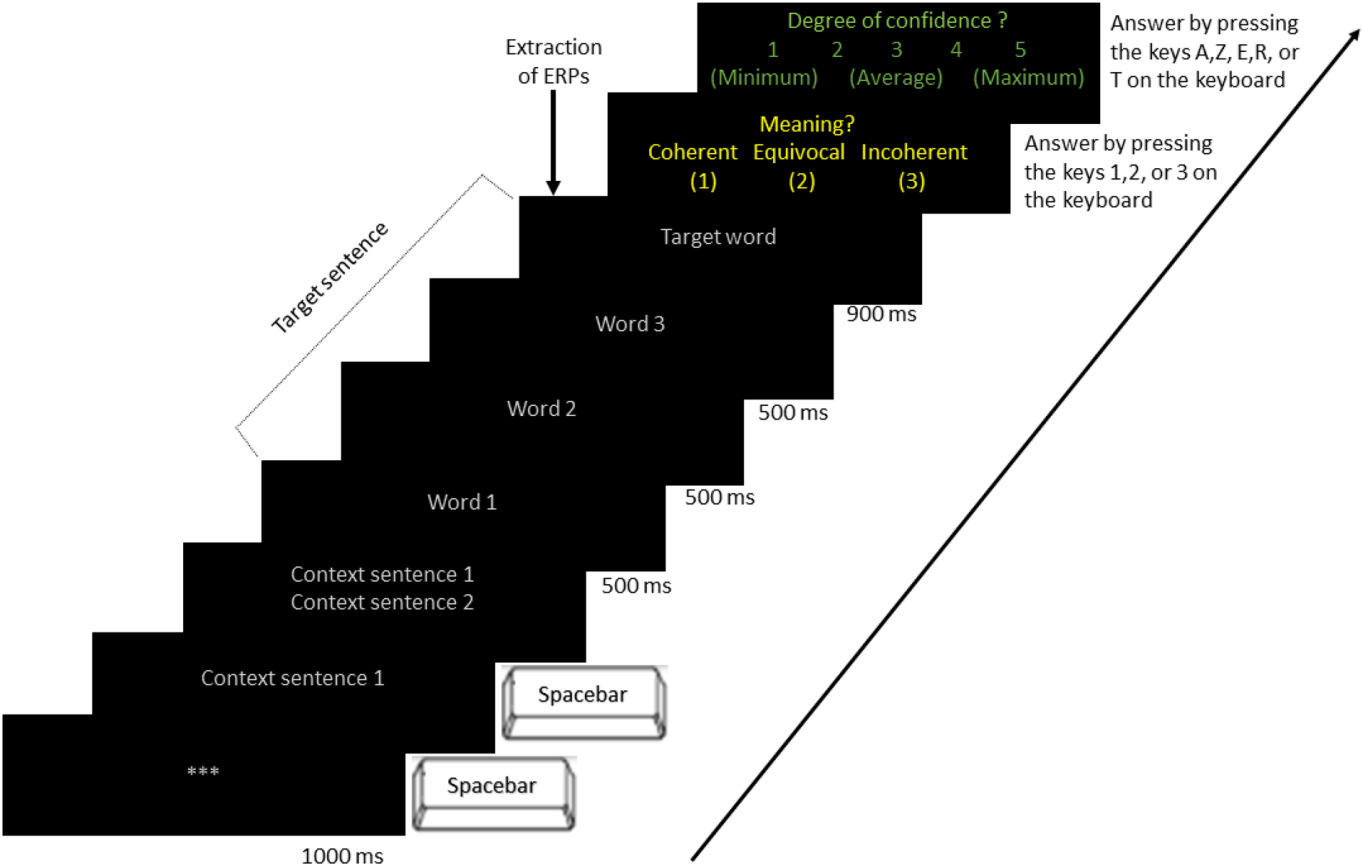
English version of the sequence of the stimuli presented to participants and to confederates with their timing. Stimuli appeared at the center of the computer screen. They responded to the questions on the screen by pressing a key whereas the confederate did so on paper (see text).

The trials were presented on a computer screen by the E-Prime software^56^. As illustrated in Fig. 2 below, each trial started with three asterisks appearing at the center of the screen for 100 ms. They were followed by the first context sentence, written with 25 pt-Arial-font letters of a grey color on a black background. The participant was asked to press the spacebar to bring up the second context sentence, which was written in the same way, and which appeared below the first context sentence for 500 ms. The participant had to press the space bar again to suppress these two sentences and to bring up the last target sentence, which was presented in the same way, but word by word. Each of these words appeared for 500 ms except the last one, which remained on the screen for 900 ms. Following this, the question word: Meaning? (“Sens?” in French) appeared on the screen with the 3 response options written in yellow 25 pt-Arial-font on a black background below the question word. The options were coherent, equivocal, and incoherent (“Cohérent”, “Équivoque” & “Incohérent” in French). Following the appearance of this option- window, the participant had to choose one of them as quickly as possible by pressing the keys 1, 2 or 3 on the keyboard. The allocation of keys 1 and 3 to the coherent and the incoherent response, respectively, was counterbalanced across participants. Key 2 was always for the equivocal response.

After the participant’s response, a question phrase, Degree of confidence? (“Degré de confiance?” in French), appeared at the center of the screen in green 25 pt-Arial-font on a black background. The participant had to assess the level of confidence in their previous response using a 5-point Likert scale. This scale appeared below the question. The participant answered using the keyboard keys Q, W, E, R or T (i.e., A, Z, E, R or T on a French keyboard) where green dots labeled 1, 2, 3, 4 or 5 were stuck, respectively. The confederate was asked to tick the boxes corresponding to her/his answers on a grid of options on paper.

### Data acquisition

Behavioral responses were collected. The EEG was recorded from the participants’ scalp using 23 electrodes inserted in a cap from EasyCap (Germany^57^) according to the extended international 10/20 system^58^ at F3/4, FC5/6, FC1/2, T7/8, C3/4, CP5/6, CP1/2, P7/8, P3/4, O1/2, Fz, Cz, and Pz sites. Linked earlobes were used as the reference. Horizontal and vertical eye movements and blinks were monitored by examining F7/8 and Fp1/2, respectively, as in^59–61^. Impedances were maintained below 10 kΩ. The EEG was amplified 10,000 times with a QuickAmp amplifier^62^. The signal was recorded continuously with a band-pass filter of 0.01–100 Hz (half amplitude cut-offs) and digitized at a sampling rate of 500 Hz.

### Data processing and measures

Continuous EEG was processed using the EEGLAB toolbox on the Matlab 2019b platform^63^. The EEG signal was divided into epochs of a 1200 ms duration, starting 200 ms before the onset of the last word of the third sentence, the story ending. EEG epochs were placed on the baseline by computing the mean voltage in the − 200 to 0 ms time window and by subtracting this mean to each of the points of the entire epochs. Epochs with excessive artefacts due to eye movements and myogram were then deleted by removing those where voltages were out of the ± 100 µV range at F7 or F8 or at Fp1 or Fp2 or out of the ± 75 µV range at any of the other 23 electrodes. Epochs with amplifier saturations or analog to digital clippings were removed by deleting trials including one or more flat lines lasting longer than 100 ms. Participants having less than 25 accepted trials in at least one of the three conditions (coherent, incoherent or equivocal) were discarded. This left 51 alones and 50 PwCs. Out of 60, the mean numbers of accepted trials in alones were 45.4 (SD 8.7), 46.2 (SD 9.8) and 43.7 (SD 9.3) for the coherent, the incoherent and the equivocal condition, respectively. The mean numbers of accepted trials in PwCs were 44.0 (SD 9.3), 46.6 (SD 9.1) and 44.1 (SD 9.4) for those three conditions, respectively. ERPs were computed by averaging the remaining EEG epochs at each of the 23 electrodes. One to 5 channels (i.e., F7, F3, F4, FC1 and/or Fz) with signals that were obviously lacking ERPs were found in 8 participants. They were replaced by an average of the ERPs of the neighboring electrodes.

A centro-parietal region of interest (ROI) was chosen for the N400 and the LPP based on the scalp location of the effects found in the social N400 studies that used written words as stimuli (e.g.,^29,31^). In effect, this scalp location is similar to that of the N400 effect classically obtained between coherent/predicted and incoherent/unpredicted written words (e.g.,^64,65^). The ROI used here thus comprised C3/4, CZ, CP1/2, CP5/6, P3/4, P7/8 and PZ electrodes. The ERP measure used was the average of the mean voltages of the ERPs at all these electrodes in the N400 (300–500 ms) and in the LPP (500–800 ms) time-window for each participant and for each of the three conditions.

Signal-to-noise ratios (SNRs) were computed as in^66,67^. The average of the mean of the ERP voltages computed at each of the ROI electrodes was used as the signal, whereas the noise was measured by the standard measurement error (the SME, as in^66^). Accordingly, in the N400 time-window, the alones had average SNRs of 2.9 (SD 2.0), 1.9 (SD 1.9), and 2.7 (SD 1.8) for the coherent, the incoherent, and the equivocal conditions, respectively. In the LPP time-window, they were 4.5 (SD 2.6), 4.08 (SD 2.4), and 5.0 (SD 2.6). In the N400 time-window, SNRs for these three conditions in PwCs were 3.0 (SD 2.0), 1.9 (SD 1.8), and 3.1 (SD 2.1). In the LPP time-window, these values were 4.5 (SD 2.4), 3.9 (SD 2.2), and 5.1 (SD 2.6), respectively.

To detect early differences (as in^20,21^), mean voltages of ERPs were also measured in the 100–200 and in the 200–300 ms time window at all electrodes.

### Analyses

An omnibus mixed-model repeated-measure ANOVA with a multivariate approach was used for the analysis of response accuracies. It had group (alones vs. PwCs) as a between-subject factor and condition (coherent vs. incoherent vs. equivocal) as a within subject factor. Another such ANOVA was used for confidence ratings.

For ERPs, two separate repeated-measure ANOVAs were conducted, one for each of the N400 and the LPP time-windows. These tests were run on the averages of the mean voltages of the ERPs of all the ROI electrodes. Each ANOVA had group as the between-subject factor with 2 levels (alones vs. PwCs) and condition as the within-subject factor with 3 levels (coherent vs. incoherent vs. equivocal). The Greenhouse–Geisser's procedure^68^ was used to compensate for variance heterogeneity across the conditions. In that case, the original F values and degrees of freedom are provided together with the corrected p-values.

As the condition factor was found to have a significant effect at each of the two time-windows, Bonferroni corrected^69^ pairwise post-hoc comparisons were used to determine the pairs of conditions that significantly differed from each other.

Additionally, two separate repeated-measures ANOVAs were run for two early time-windows, the 100–200 and the 200–300 ms ones, to detect effects occurring during the early processing of the stimulus. In each of these ANOVAs, group was the between-subject factor whereas condition and electrode were the two within-subject factors.

## Results

### Behavioral data

Behavioral data were similar across the two groups of participants (see Table 3 below). The mean response accuracy (RA) of the alones and the PwCs was 79.49% (SD 7.45). The mean confidence rating (CR) was 4.44 out of 5 (SD 0.56).

**Table 3:** Behavioral data of the two groups of participants.

Response accuracy % (RA) and confidence rating (out of 5, CR) of the three conditions	Alones Mean (SD)	Participants with a confederate (PwCs) Mean (SD)
RA coherent	83.6% (8.6)	84.5% (8.3)
CR coherent	4.5 (0.4)	4.5 (0.4)
RA equivocal	75.7% (15.1)	76.7% (10.6)
CR equivocal	4.5 (0.4)	4.5 (0.3)
RA incoherent	76.6% (13.3)	79.9% (10.7)
CR incoherent	4.4 (0.4)	4.4 (0.4)

The results of the ANOVAs performed to analyze RAs and CRs are displayed in Supplementary Table S1. They do not reveal any group effect, nor any interaction with this factor Nevertheless, as expected, RAs were higher for coherent story endings (p < 8 × 10^–7^).

### Electrophysiological data

**Figure 3:**
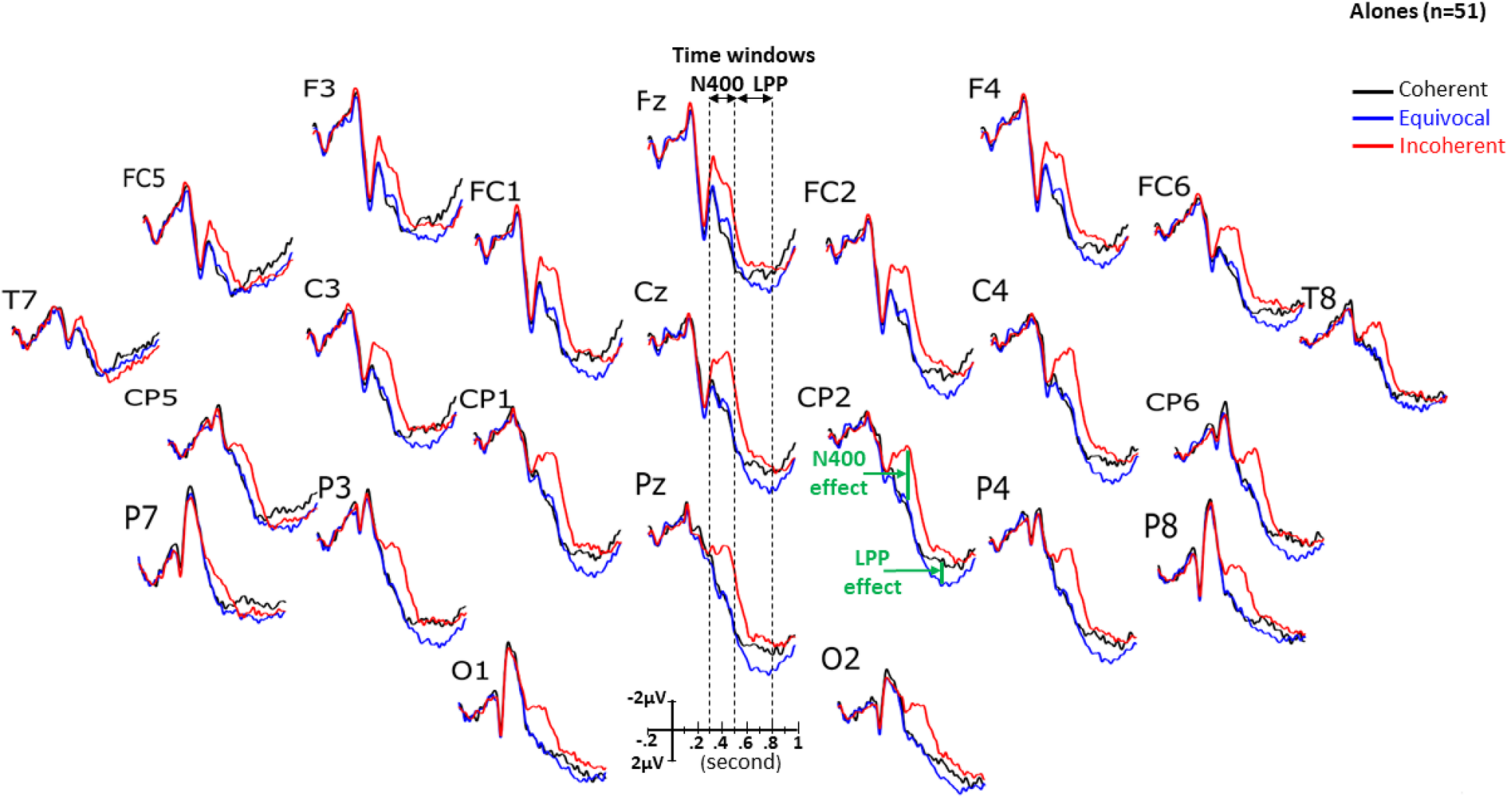
Grand averages of ERPs (n = 51) elicited by the target stimulus, that is by the last word of each short story in alone participants. Black lines are for the coherent condition, blue lines for the equivocal one and red lines are for the incoherent one.

**Figure 4:**
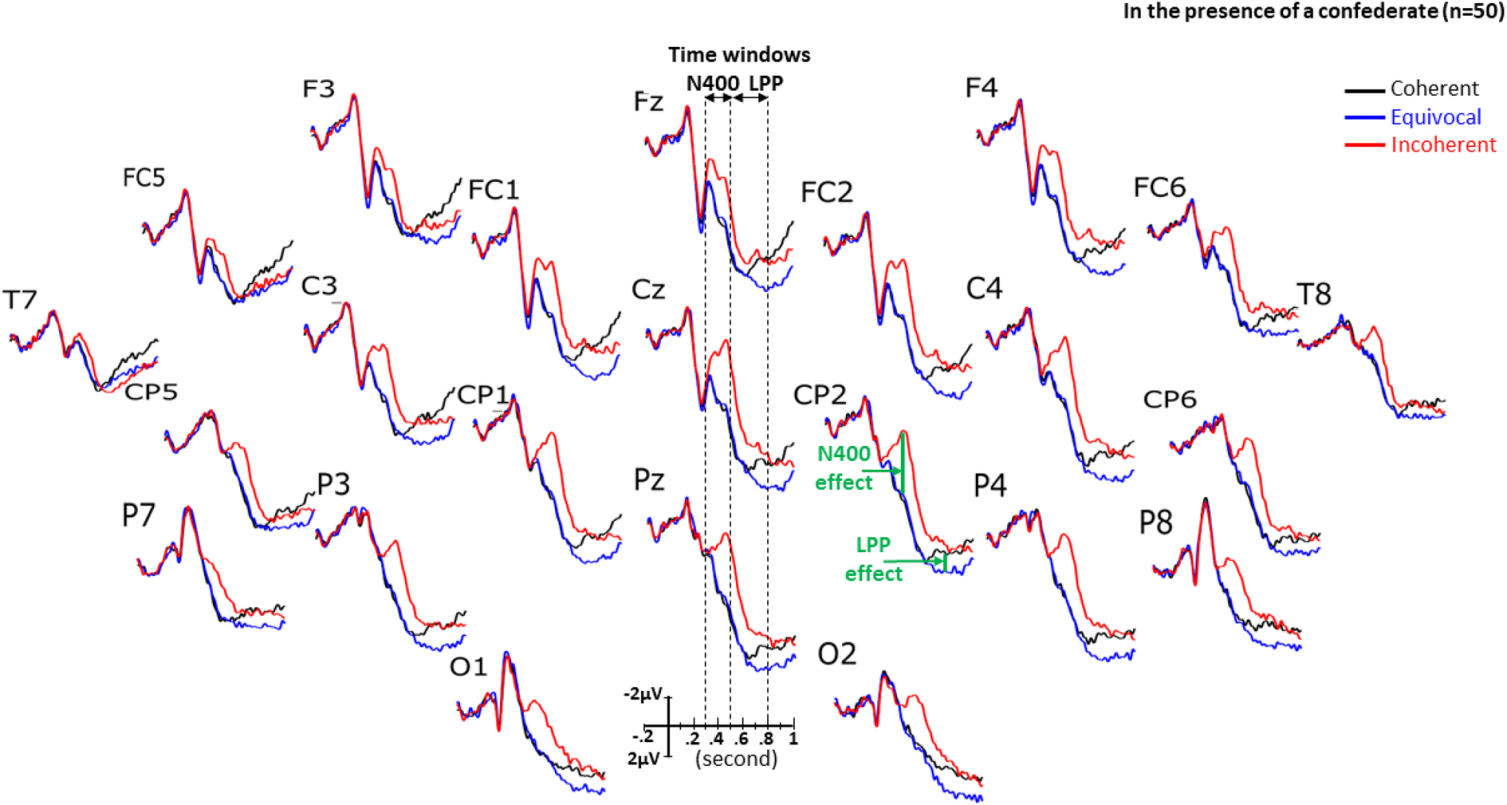
Grand averages of ERPs (n = 50) elicited by the target stimulus, that is by the last word of each short story in participants with a confederate (PwCs). Black lines are for the coherent condition, blue lines for the equivocal one and red lines are for the incoherent one.

**Figure 5:**
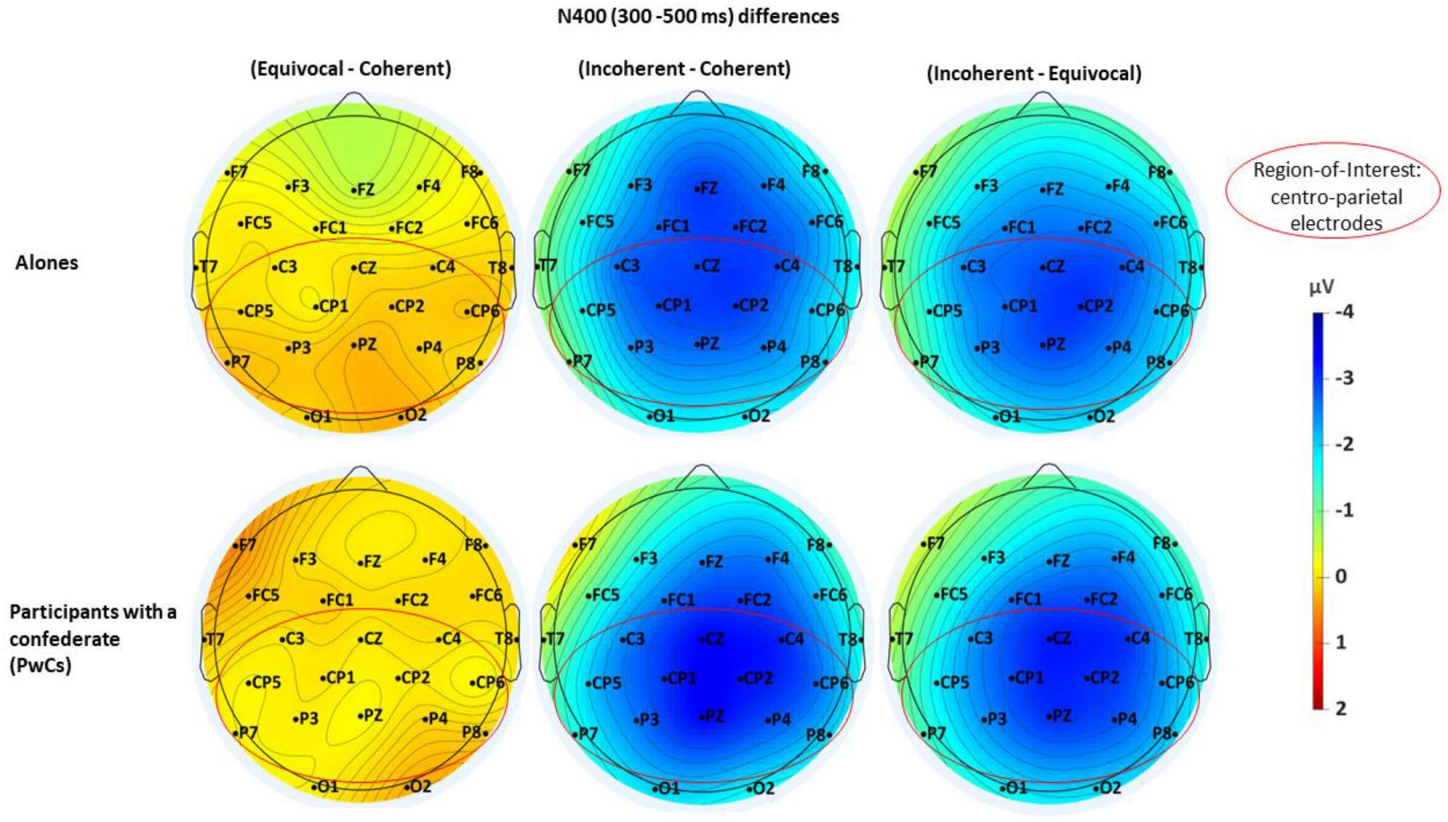
Spline interpolated iso-voltage maps of subtractions of one condition from another made in the N400 time-window (300–500 ms). The first row pictures the differences in the participants who were alone (n = 51) and, the second, differences in those who were with a confederate (PwCs, n = 50). The maps show that alones and PwCs display similar scalp distribution with the largest N400 effect between the incoherent- and the coherent-condition. The red circles mark the region-of-interest that was selected. It includes the 12 centro-parietal electrodes (C3/4, CZ, CP1/2. CP5/6, P3/4, P7/8 and PZ).

**Figure 6:**
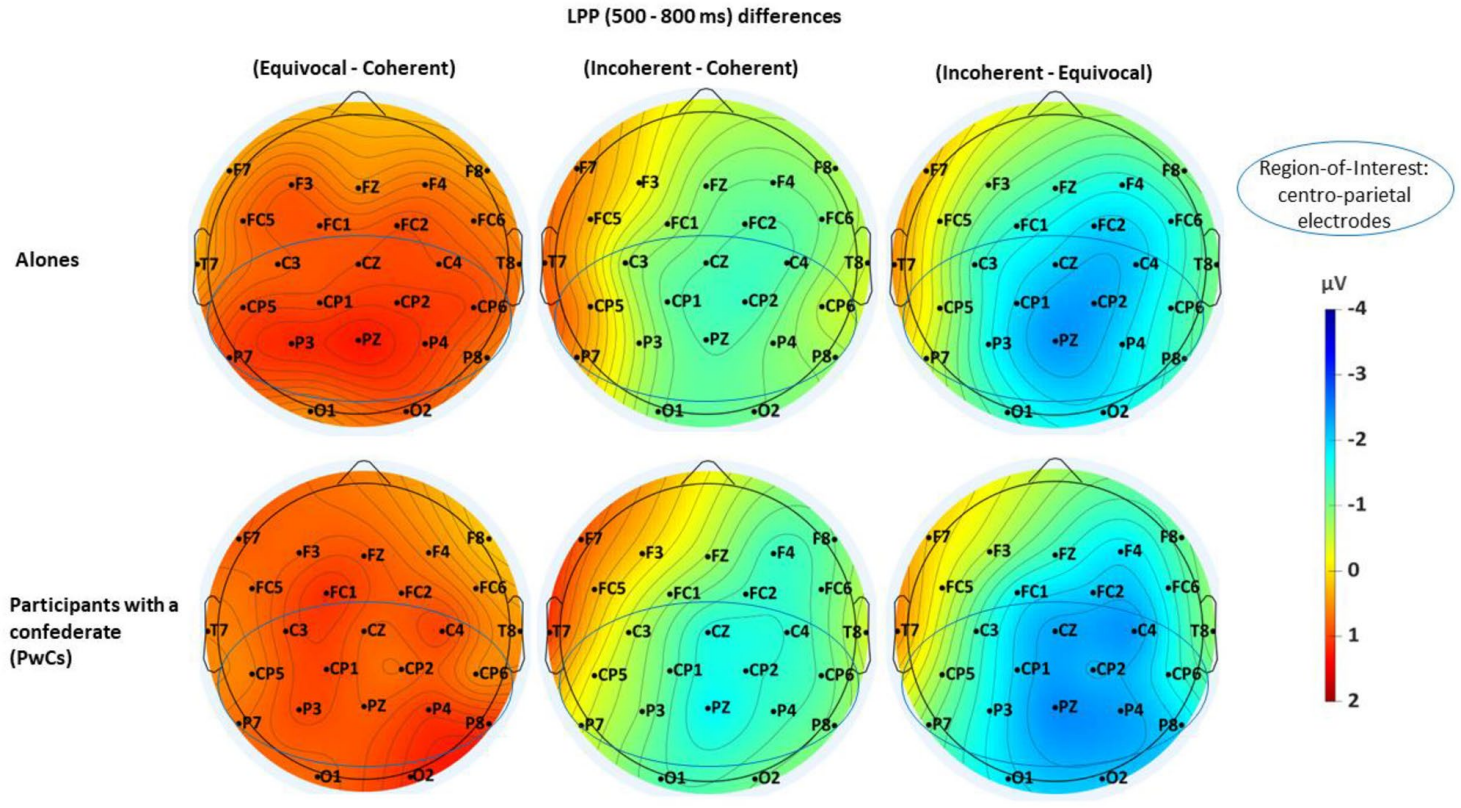
Spline interpolated iso-voltage maps subtractions of one condition from another made in the LPP time-window (500–800 ms). The first row pictures the differences in the participants who were alone (n = 51) and, the second, the differences in those who were with a confederate (PwCs, n = 50). The maps show that alones and PwCs display similar scalp distribution with the largest LPP effect between the equivocal- and the coherent-condition. The blue circles mark the selected region-of-interest. It includes the 12 centro-parietal electrodes (C3/4, CZ, CP1/2. CP5/6, P3/4, P7/8 and PZ).

At visual inspection, the grand averages of the ERPs of the alones (Fig. 3, above) appeared very similar to those of the PwCs (Fig. 4, see also Supplementary Fig. S2). On the other hand, in both groups, coherent and equivocal stimuli elicited similar ERPs in the N400 time window whereas they appear more positive in the LPP time window for equivocal than for coherent stimuli. Figure 5 displays the maps of the N400-effects in alones and in PwCs while Fig. 6 depicts those of the LPP-effects of each of these two groups. The same voltage scale was used for all scalp maps to facilitate group comparisons.

None of the two repeated-measures ANOVAs, that run with N400- and that run with LPP-measures, revealed a main effect of group nor any interaction with this factor (Table 4). In contrast, a main effect of condition was found in each of these two time-windows (Table 4), which was deconvolved by pairwise comparisons (Table 5). They revealed that N400 effects were due to differences between the incoherent and the coherent, and between the incoherent and the equivocal conditions. In the LPP time-window, significant differences were found for each of the three pairs of conditions.

**Table 4:** Results of the ANOVAs run with the average, across ROI electrodes (C3/4, CZ, CP5/6, CP1/2, P7/8, P3/4 and PZ), of the mean voltages of the ERPs in the N400 (300–500 ms) and LPP (500–800 ms) time-windows.

Time-windows	Factors Group (G, 2 levels) Conditions (C, 3 levels)	df	F-values	p-values (Greenhouse–Geisser)	Effect size (η_p_^2^)	Observed power (for an alpha = 0.05)
N400	G	1, 99	0.8	0.80	0.001	0.06
	**C**	2, 198	98	2.3 × 10^–**29**^	0.50	1.00
	G × C	2, 198	0.2	0.78	0.002	0.10
LPP	G	1, 99	0.02	0.89	1.8 × 10^–**4**^	0.05
	**C**	2, 198	26	2.2 × 10^–**10**^	0.21	1.00
	G × C	2, 198	0.2	0.77	0.002	0.1

**Table 5:** Results of the post-hoc pairwise comparisons decomposing the main effect of condition found in the N400 and LPP time-windows and reported in Table 4 above.

Time-windows	Condition pairs (Coh: coherent; Equi: equivocal; Incoh: incoherent)	Bonferroni-corrected p-values	Effect size (η_p_^2^)	Observed power (alpha = 0.05)
N400	Coh vs. Equi	1.000	0.002	0.07
	Coh vs. Incoh	3.7 × 10^–19^	0.57	1.00
	Equi vs. Incoh	1.0 × 10^–20^	0.60	1.00
LPP	Coh vs. Equi	0.001	0.12	0.95
	Coh vs. Incoh	0.001	0.12	0.96
	Equi vs. Incoh	9.5 × 10^–10^	0.33	1.00

Similarly, the repeated-measures ANOVAs run with the ERP measures in the two early time-windows, 100–200 ms and 200–300 ms neither revealed a main group effect nor any interaction with this factor (Tables 6 and 7).

**Table 6:** Results of the ANOVAs run with the mean voltages of the ERPs across all electrodes in the 100–200 ms time-window.

Factors Group (G, 2 levels) Conditions (C, 3 levels) Electrode (E, 25 levels)	df	F-values	p-values (Greenhouse–Geisser)	Effect size (η_p_^2^)	Observed power (alpha = 0.05)
G	1, 99	0.2	0.64	0.002	0.08
C	2, 198	0.4	0.68	0.004	0.11
E	24, 2376	9.9	4.6 × 10^–5^	0.1	0.99
G × C	2, 198	1.5	0.23	0.02	0.31
G × E	24, 2376	0.5	0.18	0.01	0.41
C × E	48, 4752	1.5	0.15	0.02	0.64
G × C × E	48, 4752	0.7	0.65	0.01	0.31

**Table 7:** Results of the ANOVAs run with the mean voltages of the ERPs across all electrodes in the 200–300 ms time-window.

Factors Group (G, 2 levels) Conditions (C, 3 levels) Electrode (E, 25 levels)	df	F-values	p-values (Greenhouse–Geisser)	Effect size (η_p_^2^)	Observed power (alpha = 0.05)
G	1, 99	0.2	0.63	0.002	0.08
C	2, 198	3.0	0.05	0.03	0.58
E	24, 2376	140	2.2 × 10^–48^	0.6	1.00
G × C	2, 198	0.5	0.62	0.005	0.13
G × E	24, 2376	1.7	0.18	0.02	0.40
C × E	48, 4752	4.1	2.0 × 10^–4^	0.04	0.99
G × C × E	48, 4752	0.8	0.61	0.01	0.33

## Discussion

In this study, we looked at whether the increase in N400 amplitude that can be induced by the presence of another person also occurs when that person is not in sight. This person was placed a little behind participants who knew for sure that (s)he was there during the entire experiment. As in two recent social N400 studies^44,45^, participants could see that this other person was getting the same stimulus information as they were. They also knew that (s)he was performing the task, for which they both had to decide whether the word that ended each of the short stories presented was coherent, incoherent or equivocal. Although the classical N400-effect was found between the incoherent and the coherent conditions, no N400 increase was found in the participants who were with the confederate (the PwCs, n = 50) relative to those who were alone (n = 51), not in any of the three conditions. These results thus showed that the mere knowledge of the confederate’s presence during the entire experiment is not sufficient to induce a social N400-increase. They suggest that the confederate has to remain in sight of the participant so that at least some visual interaction exists.

This lack of N400 difference between the PwCs and the alones, even in the easiestcondition, that is, in the coherent one, suggests that the PwCs did not attempt to adopt also the perspective of the confederate and/or to build a commun ground. They would have focused on their own perspective to process the stimuli, as if they were alone. These findings thus differ from those collected by Forgacs’s team^45^ in the absence of a task instruction and from those of Hinchcliffe et al.’s work^44^ where participants performed a simple sentence-correctness task. There, the presence of a confederate, who was facing participants or was in the periphery of their visual field, respectively, induced a social N400-increase. Taken together, these literature results and those of the present study suggest that the automatic perspective-taking requires not only on the knowledge of the presence of someone else, but also a form of interaction with that person, like the visual one that existed in those two recent literature studies.

On the other hand, the amplitudes of the N400s elicited by equivocal endings were found to be similar to those of the N400s to coherent endings, as already observed in previous studies^47–49^. This can neither be explained by the N400-activation-, nor by the N400-integration-difficulty-hypothesis mentioned in the introduction. In effect, according to the activation idea, the N400s evoked by equivocal endings should have been larger than those evoked by the coherent ones because equivocal story endings were less predictable than the coherent endings. Their mean cloze probability was only 0.21 whereas that of the coherent endings was 0.53. Therefore, more semantic activation remained to be performed for the former than for the latter. The similarly small N400s of coherent and equivocal endings are not consistent with the N400-integration hypothesis either. In effect, this hypothesis would have predicted larger N400s for equivocal than for coherent endings since the richer the semantics, the greater the difficulty in integrating it into their context. Finally, given that more adjustment should be needed for less predicted words, it seems that equivocal endings should also have evoked larger N400s than coherent endings according to predictive-error models (e.g.,^26,27^).

In contrast, this absence of N400 difference between equivocal and coherent might be consistent with the N400-inhibition hypothesis. In effect, this hypothesis predicts that the N400 should *not* be larger if no additional semantic inhibition is performed (e.g.,^36–38,40^). This should be the case here since semantic richness and low predictability in themselves do not necessitate any additional amount of inhibition. As a matter of fact, this would be particularly unneeded here, where equivocality was task relevant and thus when keeping multiple meanings was important.

The social N400 effect discovered by Rueschemeyer, Gardner, and Stoner^29^ and replicated by Jouravlev et al.^31^ might also be consistent with the N400-inhibition hypothesis. Indeed, taking the confederate's perspective into account as well could only be done by excluding, from some neural systems, the privileged information that participants had but knew the confederate was missing. A similar account can be proposed to account for the N400 increases found in Hinchcliffe et al.^44^ and Forgacs et al.’s^45^ studies. It would be based on the fact that a stimulus activates not only common representations (e.g., common to both the participant and the confederate) but also privileged information, that is, representations that exist only in the participant, such as those of the last context-episode where the participant encountered the stimulus before the experiment, for instance, and/or, such as emotions and actions associated to that stimulus for the participant (e.g., Proust's madeleine^70^). During the visual interaction, perspective taking or processes building the common ground might set aside these representations and be responsible for larger N400 amplitudes according to the N400 inhibition hypothesis.

Incidentally, in Forgacs et al.'s study^45^, the source of the auditory stimulus and the position of the visual stimulus for the confederate could also be at stake. Participants were aware that this source and this position were on the opposite hand side for the confederate they were facing (i.e., they were on the left hand side of the confederate and on the right hand side for them). Visual perspective-taking and spatial perspective-taking studies have shown that this automatically impacts participants^41,46^.

On the other hand, it must be noted that the N400 and the late posterior positivity (LPP) partially overlap. In effect, in each participant, the N400 occurs a bit later at some trials while, in others, the LPP occurs a bit earlier. Moreover, some participants have late N400s while others have early LPPs. Given that N400 and LPP have opposite electrical polarity, they diminish the raw amplitude of each other on averages and grand averages. This also reduces the size of the impacts that affect only one of them in one direction. In this context, the absence of N400 difference between the equivocal- and the coherent-condition and the presence of a significantly larger LPPs for equivocal than for coherent endings are even more striking; just as the results of the repeated-measures omnibus ANOVA done with the N400 and the LPP as the two levels of the time-window factor. In effect, they revealed a significant interaction of this factor with conditions (Supplementary Table S2).

The LPP is a potential also referred to as the P300b for simple and repeated lab stimuli and as to the P600 or to the LPC in word studies (e.g.,^71–76^). Here, in both groups, as mentioned, these potentials were found to be of larger amplitudes for equivocal story endings than for the two other types of endings (Figs. 3 and 4). This pattern suggests that, when a task requires the detection of ambiguities, like the one used in the current study, multiple meanings may be retained rather than inhibited. This is consistent with the idea that the amplitude of the LPP family of potentials is positively correlated with the amount of information placed in working memory^72^. For instance, when stimuli are presented just when there could be an attentional blink, the LPP is found to be larger for stimuli that are consciously perceived than for those that are not^43^ (unlike the N400). The LPP is also larger when more than one meaning is consciously perceived, such as when the equivocality (example: irony/sarcasm) of a word is detected^47^. Stimuli that can reactivate many memory episodes evoke larger LPPs than those that reactivate only one or a few such episodes (e.g.^7^). Finally, faces that are judged to be ambiguous elicit larger (and later) LPPs than faces that are judged to be either positively or negatively valenced^77^. Keeping in mind multiple meanings in tasks requiring the detection of ambiguity goes with lesser inhibitions. This fits the absence of N400 difference between equivocal and coherent endings predicted by the N400-inhibition hypothesis.

Lastly, the absence of perspective-taking and of common ground building indicated by the lack of social N400 increase does not rule out the possibility that these processes occur, at least partially, earlier than the N400 during the processing of the story ending. To examine this issue, a repeated-measure ANOVA was run in two early time-windows, the 100–200 and the 200–300 ms one. This analysis did not reveal any effect of group nor any interaction including this factor. However, it has to be emphasized that this does not exclude the possibility that some perspective-taking and/or common ground building occur before the appearance of the story ending and be part of the pragmatic processing that can take place at any time (for a review, see^78^).

Summing up, the results of the present study support the hypothesis that N400 indexes inhibition. They also suggest that just being aware of the presence of another person nearby (i.e., a bit behind oneself) is not sufficient to trigger the building of a common ground from which privileged information is set aside. Thereby, it adds to the literature that interaction, at least purely visual, may be important for the building of a “common ground”^34^. The absence of social N400s in our results further suggest that attributing to other’s mental states or theory of mind^79,80^ can be repressed not only by the difficulty of the task of participants but also by some social circumstances. Knowing when and in which circumstances perspective-taking occurs appears to be of critical importance. For instance, seeing whether or not social N400 effects also depend on the relationship between the participant and the other person.

### Supplementary Information

Supplementary Information.

Supplementary Information.

## Data Availability

Raw (= EEG) data of all subjects in the EEGLAB format (a MATLAB plugin), codes used to process EEGs, ERPs of each condition of each subject in the ERPLAB format (a MATLAB plugin), Excel tables of mean-voltage measures in each time-window for each subject and each condition and SPSS output files of the mixed-model ANOVAs will be available on request to the last author. J. B. Debruille will respond to all readers’ enquiries and requests for any materials. Such materials will be placed in the open science framework (https://osf.io) after acceptance of the manuscript.
