# Supplementary material for: Unveiling the need of interactions for social N400s and supporting the N400 inhibition hypothesis
Source: Sci Rep. 2023 Aug 3;13:12613. doi: 10.1038/s41598-023-39345-6 (PMC10400652; doi:10.1038/s41598-023-39345-6)
Supplement: Supplementary file 1 — Supplementary Information. [file 41598_2023_39345_MOESM1_ESM.pdf]

Supplementary Information

Unveiling the need of interactions for social N400s and supporting the N400 inhibition hypothesis

Sujata Sinha,[1,2] Sarah Del Goleto[3], Milena Kostova[3] and J. Bruno Debruille,[1,2,4] *

Affiliations:

[1]Department of Neurosciences, Faculty of Medicine, McGill University, Montréal, Canada.

[2] Research Center of the Douglas Mental Health University Institute, Montréal, Canada.

[3]UR Paragraphe, Université Paris 8 Vincennes-Saint-Denis, Saint-Denis, France.

[4]Department of Psychiatry, Faculty of Medicine, McGill University, Montréal, Canada.

* Corresponding author: bruno.debruille@mcgill.ca

Supplementary Figure S1 Consent form

Supplementary Figure S2 Grand averages of ERPs elicited by the target stimulus, that is by the last word of each short story, in alones (n = 51) versus participants with a confederate (PwCs, n = 50) for each of the three conditions.

- **Figure S2.a** Coherent condition
- **Figure S2.b** Incoherent condition
- **Figure S2.c** Equivocal condition

Supplementary Table S1 Results of the omnibus ANOVA and of the Benjamini-Hochberg FDR procedure (B-H)** for 2 groups run with the mean response accuracy percentages and the mean confidence ratings

- **Table S1.a** Results of the ANOVA on mean response accuracy percentages
- **Table S1.b** Results of the post-hoc pairwise comparisons decomposing the main effect of condition (C) in Table S2.a
- **Table S1.c** Results of the ANOVA on mean confidence-ratings
- **Table S1.d** Results of the post-hoc pairwise comparisons decomposing the main effect of condition (C) in Table S2.c

Supplementary Table S2 Results of the omnibus ANOVA and of the Benjamini-Hochberg FDR corrections (B-H)** for 2 groups run with the mean voltage of ERPs at all electrodes in the N400 (300-500 ms) and LPP (500-800 ms) time-windows

Formulaire de consentement (exemple)

Formulaire de consentement

Je soussigné donne mon accord pour participer bénévolement à une étude en neurosciences cognitives portant sur le langage, conduite par Milena Kostova et Sarah Del Goleto. J'ai bien compris qu'à l'occasion de cette étude, on me présentera de courtes histoires pour lesquelles j'effectuerai une tâche de compréhension et que je remplirai différents questionnaires. Un enregistrement EEG sera effectué pendant la réalisation de la tâche. Elle comporte une session de trois heures.

L'objectif de cette recherche concerne la compréhension des mécanismes cognitifs et cérébraux responsables de la compréhension du langage. J'ai bien noté que je suis bien évidemment libre de refuser l'étude ou de l'interrompre à tout moment. J'ai bien noté que les données enregistrées seront traitées en respectant l'anonymat et éventuellement utilisées dans le cadre d'une publication scientifique ou médicale.

Date :

PARTICIPANT A L'ETUDE :

Nom : Prénom : Signature :

RESPONSABLES DE L'ETUDE :

Milena Kostova Sarah Del Goleto

Signature :

Supplementary Figure S1 Consent form

Supplementary Figure S2 Grand averages of ERPs elicited by the target stimulus, that is by the last word of each short story, in alones (n = 51) versus participants with a confederate (PwCs, n = 50) for each of the three conditions.

Figure S2.a Coherent condition

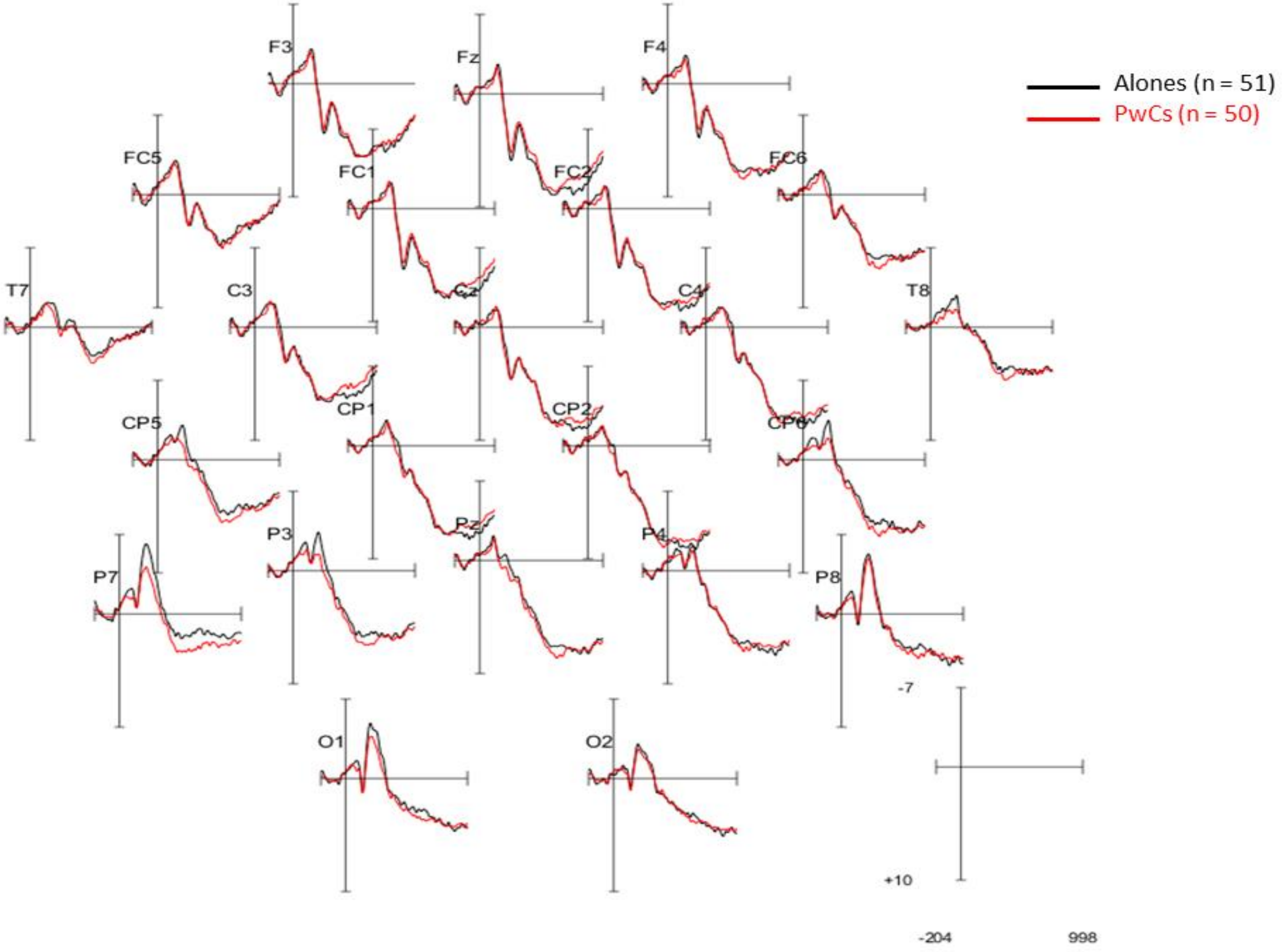

Figure S2.b Incoherent condition

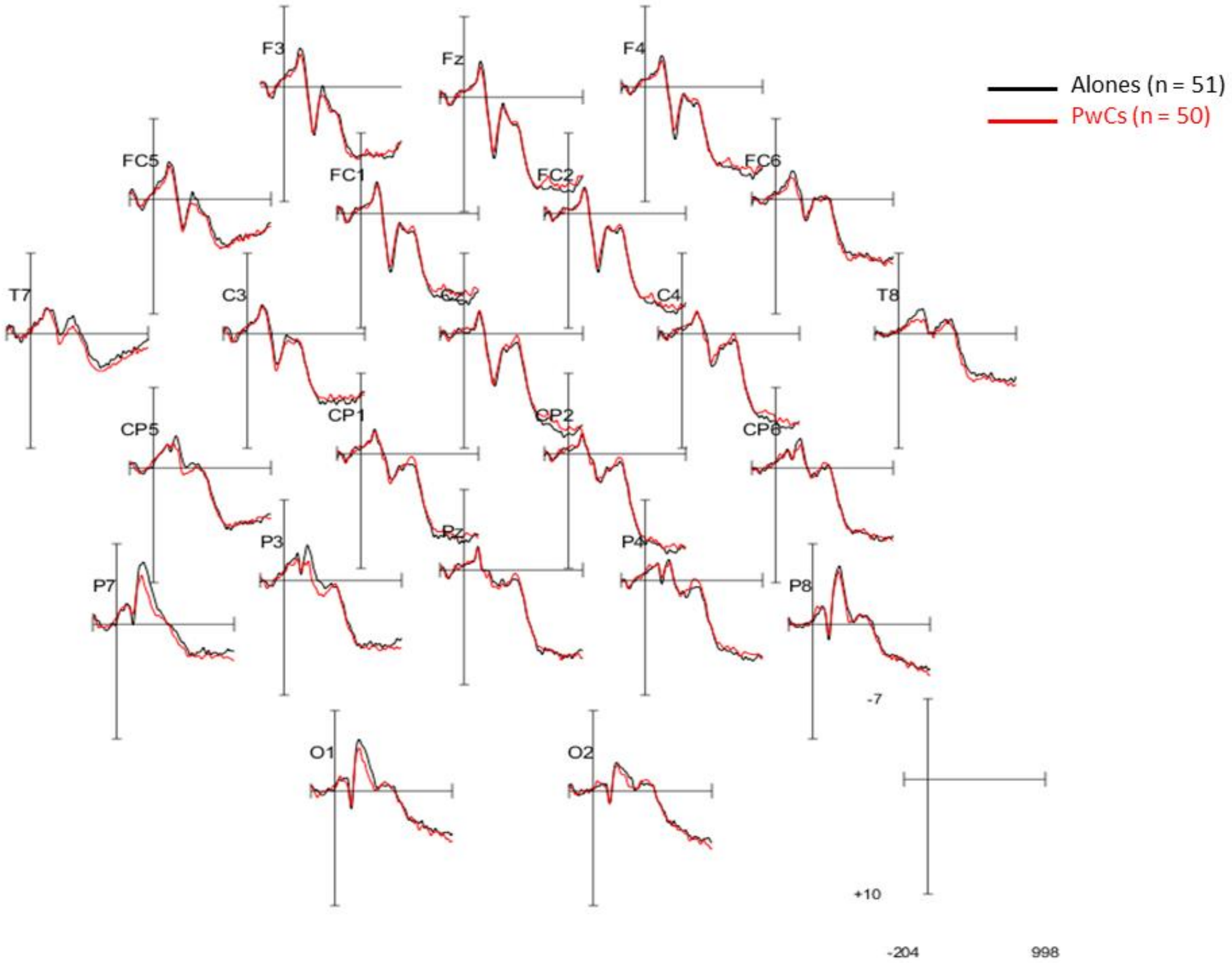

Figure S2.c Equivocal condition

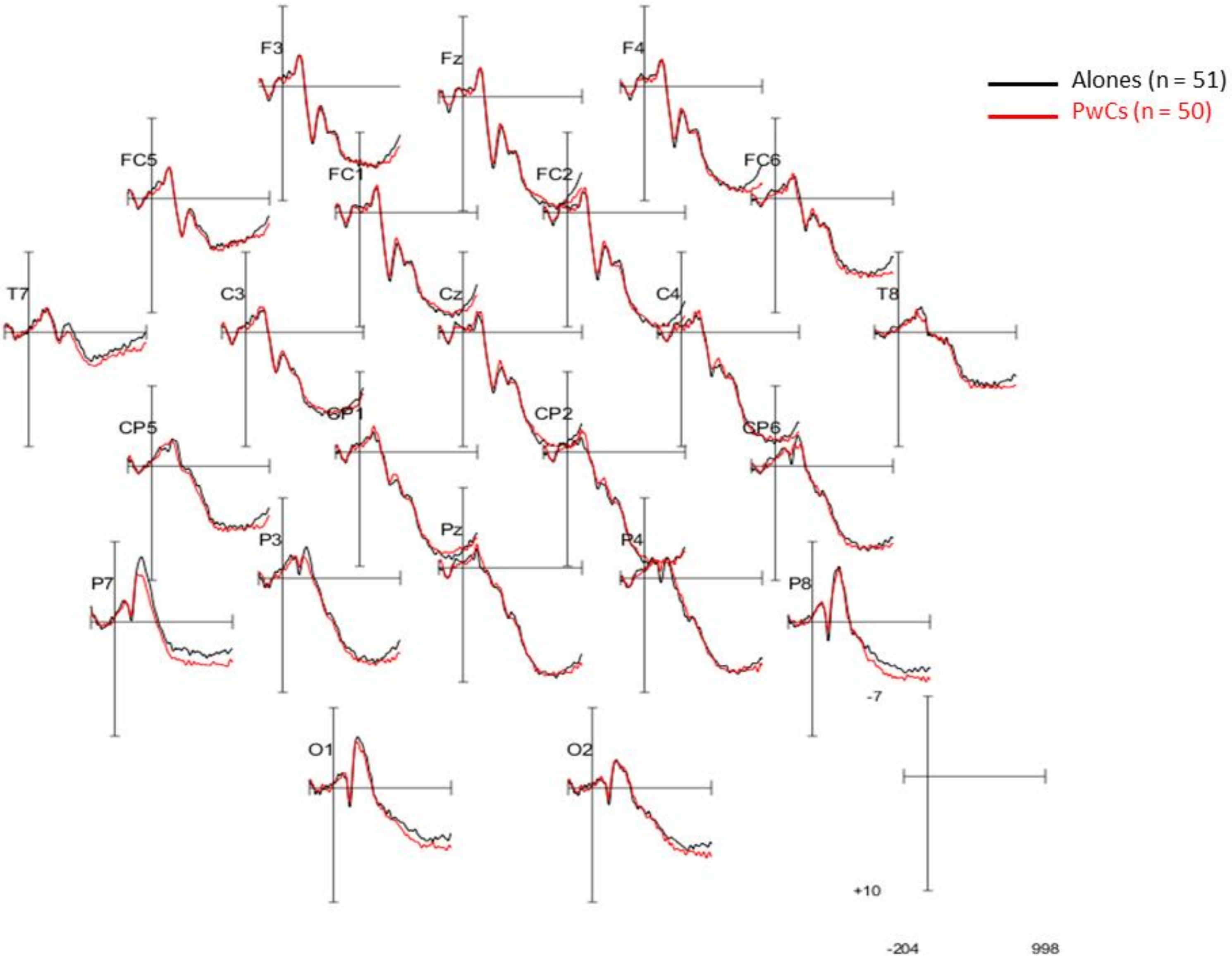

Supplementary Table S1 Results of the omnibus ANOVA and of the Benjamini-Hochberg FDR procedure (B-H)** for 2 groups run with the mean response accuracy percentages and the mean confidence ratings

Table S1.a Results of the ANOVA on mean response accuracy percentages

**Number of tests (N)**	**Factors** Group (G, 2 levels) Conditions (C, 3 levels)	**df**	**F-values**	**p-values (Greenhouse-Geisser)**	**Rank (r) of the p-value**	**Critical value = FDR*(r/N)**	**Significant according to B-H**	**Effect size (η_p^2)**	**Observed Power (alpha=0.05)**
3	G	1, 99	1.4	0.234	2	0.067	no	0.01	0.22
	C	2, 198	15.0	1.1×10^{-6}	1	0.033	yes	0.1	0.99
	G × C	2, 198	0.4	0.667	3	0.100	no	0.004	0.11

Table S1.b Results of the post-hoc pairwise comparisons decomposing the main effect of condition (C) in Table S2.a

**No of comparisons**	**Condition pairs (Coherent: Coh; Equivocal: Equi; Incoherent: Incoh)**	**p-value**	**Rank (r)of the p-value**	**Critical value = FDR * (r/N)**	**Significant according to B-H**	**Effect size (cohen's d)**
3	Coh vs. Equi	8.1×10^{-7}	1	0.033	yes	0.5
	Coh vs. Incoh	5.3×10^{-3}	2	0.067	yes	0.4
	Equi vs. Incoh	0.201	3	0.100	no	0.1

Table S1.c Results of the ANOVA on mean confidence ratings

Number of tests (N)	Factors<br>Group (G, 2 levels)<br>Conditions (C, 3 levels)	df	F-values	p-values (Greenhouse-Geisser)	Rank (r) of the p-value	Critical value = FDR*(r/N)	Significant according to B-H	Effect size (η_p^2)	Observed Power (alpha=0.05)
3	G	1, 99	0.1	0.793	3	0.100	no	0.01	0.22
	C	2, 198	17.8	5.6×10^{-7}	1	0.033	yes	0.2	0.99
	G × C	2, 198	1.3	0.270	2	0.067	no	0.01	0.26

Table S1.d Results of the post-hoc pairwise comparisons decomposing the main effect of condition (C) in Table S2.c

No of comparisons	Condition pairs (Coherent: Coh; Equivocal: Equi; Incoherent: Incoh)	p-value	Rank (r)of the p-value	Critical value = FDR * (r/N)	Significant according to B-H	Effect size (Cohen's d)
	Coh vs. Equi	1.0	3	0.100	no	0.04
3	Coh vs. Incoh	4.8×10^{-5}	2	0.067	yes	0.5
	Equi vs. Incoh	1.5×10^{-5}	1	0.033	yes	0.5

** The Benjamini-Hochberg (B-H) critical value, is equal to FDR * (r/N) where FDR is the False Discovery Rate (10%), r is the tank of the p-value when all p-values of the analysis are sorted in the ascending order, and N is the total number of tests and thus, of p-values produced, by this analysis. The p-values in bold are those that are smaller than their FDR threshold and that are thus considered as statistically significant according to the B-H procedure.

Supplementary Table S2 Results of the omnibus ANOVA and of the Benjamini-Hochberg FDR corrections (B-H)** for 2 groups run with the mean voltage of ERPs at all electrodes in the N400 (300-500 ms) and LPP (500-800 ms) time-windows

**No. of tests** (N)	**Factors** Group (G, 2 levels) Time-window (T, 4 levels) Conditions (C, 3 levels) Electrode (E, 25 levels)	**df**	**F-values**	**p-values** (Greenhouse-Geisser)	**Rank (r) of the p-value**	**Critical value = FDR * (r/N)**	**Significant according to B-H**	**Effect size** (η_p^2)	**Observed Power** (alpha=0.05)
	G	1, 99	0.8	0.780	12	0.080	no	0.001	0.06
15	**T**	1, 99	235.6	**6.2×10^{-28}**	3	0.020	**yes**	0.7	1.00
	C	2, 198	52.9	1.2×10^{-18}	5	0.033	yes	0.3	1.00
	E	24, 2376	117.7	4.3×10^{-64}	1	0.007	yes	0.5	1.00
	G × T	1, 99	0.01	0.946	14	0.093	no	4.6×10^{-5}	0.05
	G × C	2, 198	0.05	0.947	15	0.100	no	0.001	0.06
	G × E	24, 2376	1.4	0.233	8	0.053	no	0.01	0.43
	T × C	2, 198	35.2	3.6×10^{-13}	6	0.040	yes	0.3	1.00
	T × E	24, 2376	59.3	7.9×10^{-29}	2	0.013	yes	0.4	1.00
	C × E	48, 4752	19.5	1.7×10^{-26}	4	0.027	yes	0.2	1.00
	G × T × C	2, 198	0.1	0.905	13	0.087	no	0.001	0.06
	G × T × E	24, 2376	0.5	0.696	11	0.073	no	0.005	0.14
	G × C × E	48, 4752	0.9	0.484	9	0.060	no	0.01	0.44
	T × C × E	48, 4752	7.7	2.9×10^{-8}	7	0.047	yes	0.07	1.00
	G × T × C × E	48, 4752	0.7	0.617	10	0.067	no	0.007	0.31

** The Benjamini-Hochberg (B-H) critical value, is equal to FDR * (r/N) where FDR is the False Discovery Rate (10%), r is the rank of the p-value when all p-values of the analysis are sorted in the ascending order, and N is the total number of tests and thus, of p-values produced, by this analysis. The p-values in bold are those that are smaller than their FDR threshold and that are thus considered as statistically significant according to the B-H procedure.
