# Supplementary material for: Unveiling the need of interactions for social N400s and supporting the N400 inhibition hypothesis
Source: Sci Rep. 2023 Aug 3;13:12613. doi: 10.1038/s41598-023-39345-6 (PMC10400652; doi:10.1038/s41598-023-39345-6)
Supplement: Supplementary file 2 — Supplementary Information. [file 41598_2023_39345_MOESM2_ESM.docx]

Supplementary material

Kutas and Federmeier, in their 2011-work^6^, went against this N400 inhibition idea. They based their argument on the large N400s found in cases where contexts do not allow precise predictions. These large N400s are evoked by unpredictable words (e.g., “wind”) when the sentence frame at the end of which they occurred (e.g., “He was soothed by the gentle...”) do not allow the activation of one particular ending^82^. Thus, for Kutas and Federmeier^6^, if the N400 inhibition hypothesis were correct, words occurring after such contexts should elicit smaller N400s because no inaccurate predictions have to be inhibited. In contrast with this prediction, N400s are approximately as large as those elicited by unpredictable words (e.g., “dog”) occurring after sentence frames (e.g., “Don’t touch the wet...”) that activate a precise ending (e.g., “paint”).

Nevertheless, this argument can be rebutted. Low constraining sentence frames, such as “He was soothed by the gentle...”, activate in a shallow way several possible endings (i.e., the endings that are produced in the sentence completion task of Bloom and Fishler’s experiment^83^, that is: “breeze”, “music”, “touch”, “massage”, “hands”, “nurse” etc.). The total amount of these shallow activations might be similar to the total amount of activation induced by a sentence frame constraining towards only one ending. The amount of inhibition of inaccurate predictions to be performed could then be similar, accounting for the similarly large N400 amplitudes. This similarity of the N400 amplitude in the two cases may thus not go against the idea that part of the N400 indexes the inhibition of inaccurate predictions.
